# Multiple imputation and direct estimation for qPCR data with non-detects

**DOI:** 10.1186/s12859-020-03807-9

**Published:** 2020-11-26

**Authors:** Valeriia Sherina, Helene R. McMurray, Winslow Powers, Harmut Land, Tanzy M. T. Love, Matthew N. McCall

**Affiliations:** 1 Department of Biostatistics and Computational Biology, University of Rochester Medical Center, 265 Crittenden Blvd., 14642 Rochester, NY USA; 2 Department of Biomedical Genetics, University of Rochester Medical Center, 601 Elmwood Ave., 14642 Rochester, NY USA; 3 Department of Pathology and Laboratory Medicine, University of Rochester Medical Center, 601 Elmwood Ave., 14642 Rochester, NY USA; 4 Department of Biomedical Engineering, University of Rochester, 201 Robert B. Goergen Hall, 14627 Rochester, NY USA

**Keywords:** Gene expression, Quantitative real-time PCR (qPCR), Missing not at random (MNAR), Non-detects, Direct estimation, Multiple imputation

## Abstract

**Background:**

Quantitative real-time PCR (qPCR) is one of the most widely used methods to measure gene expression. An important aspect of qPCR data that has been largely ignored is the presence of non-detects: reactions failing to exceed the quantification threshold and therefore lacking a measurement of expression. While most current software replaces these non-detects with a value representing the limit of detection, this introduces substantial bias in the estimation of both absolute and differential expression. Single imputation procedures, while an improvement on previously used methods, underestimate residual variance, which can lead to anti-conservative inference.

**Results:**

We propose to treat non-detects as non-random missing data, model the missing data mechanism, and use this model to impute missing values or obtain direct estimates of model parameters. To account for the uncertainty inherent in the imputation, we propose a multiple imputation procedure, which provides a set of plausible values for each non-detect. We assess the proposed methods via simulation studies and demonstrate the applicability of these methods to three experimental data sets. We compare our methods to mean imputation, single imputation, and a penalized EM algorithm incorporating non-random missingness (PEMM). The developed methods are implemented in the R/Bioconductor package nondetects.

**Conclusions:**

The statistical methods introduced here reduce discrepancies in gene expression values derived from qPCR experiments in the presence of non-detects, providing increased confidence in downstream analyses.

## Background

Polymerase chain reaction (PCR) uses short-length oligonucleotide *primers* to initiate and direct synthesis of new DNA copies using DNA polymerase plus single-stranded DNA as a template [1]. Oligonucleotides complementary to each of the two possible sequences relating to the sense and anti-sense strands of the target DNA are included in the reaction, allowing both strands to be amplified simultaneously. These new DNA copies are added to the pool of DNA templates and the process is repeated multiple times, so that amplification occurs by chain reaction [2]. The use of fluorescent tags allows one to quantify the amount of DNA present at each PCR cycle; this is referred to as *quantitative PCR* or *qPCR*. In addition to quantifying DNA, qPCR can be used to measure gene expression at the level of mRNA by first generating a complementary DNA (cDNA) via reverse transcription of the pool of mRNA and then using the cDNA for target amplification by PCR.

Despite continuing advances in genomic technology, qPCR remains the gold standard for measuring gene expression below genome-scale [3]. Given the high sensitivity and relatively simple wet-lab protocol, qPCR has been adapted for a range of uses from measuring expression of a single gene to the simultaneous measurement of thousands of genes in the same sample. Unlike other genomic technologies, qPCR allows one to measure the rate of amplification across PCR cycles. Analysis of the resulting amplification data across PCR cycles can be used to estimate a quantity proportional to the initial amount of DNA in a sample, referred to as the quantification cycle (Cq) [3]. This quantity is inversely proportional to the number of target molecules in the initial pool; therefore, a higher Cq value implies there was lower expression of the target in a sample. Cq values are either related to a known set of copy number standards or a control gene (absolute quantification) [4, 5] or to the Cq value of the same target in another sample (relative quantification) [6].

An important issue in qPCR experiments that has been largely ignored is the presence of non-detects, those reactions failing to produce a Cq value. This is especially a concern in highly multiplexed qPCR experiments where a small minority of reactions represent non-detects and simply repeating the experiment to try to obtain those few missing values is not feasible. For threshold-based quantification methods, these missing values occur when an amplification does not exceed the predetermined threshold. Model-based quantification methods [7, 8] often require an exponential and plateau phase to accurately quantify the target. If non-detects occurred completely at random, then simply removing them would lead to unbiased and consistent estimates of expression. Alternatively, if non-detects were missing at random given the expression in replicate samples, a mean imputation procedure, in which missing values are replaced by their conditional expectation, would produce unbiased and consistent estimates. However, this approach would distort the distribution of gene expression and lead to underestimation of residual variance [9].

Previously, we showed that the probability of a non-detect increases as the expression of the target transcript decreases; therefore, non-detects do not occur completely at random [10]. While it is often not possible to distinguish between missing at random and missing not at random from the observed data, specific aspects of the technology and prior analysis of a large control data set suggested that qPCR non-detects are likely missing not at random. This results in an uneven distribution of non-detects across both genes and samples, such that lowly expressed genes are more likely to produce non-detects as are samples with overall lower signal. We previously developed a single imputation (SI) procedure that treats non-detects as non-random missing data, models the missing mechanism as a monotone increasing function of gene expression, and uses an Expectation Maximization (EM) algorithm to impute missing values [10]. This approach was a significant improvement over previous approaches and was shown to improve the predictive accuracy of a biomarker of prostate cancer recurrence [11]. However, by replacing missing values with imputed values, the SI procedure underestimates the residual variance, leading to anti-conservative inference.

We address this limitation by developing two new methods to handle qPCR non-detects: (1) direct estimation of the mean and variance of gene expression using maximum likelihood estimation (DirEst) and (2) a multiple imputation (MI) procedure that models three sources of variability: uncertainty in the missing data mechanism, uncertainty in the parameter estimates, and measurement error. Our model of the missing data mechanism arises from the biochemical processes central to qPCR experiments. Additionally, we introduce models for both absolute and relative quantification of gene expression; the latter allows one to adjust for potential batch effects. Finally, we compare these methods to our previous single imputation method, a simple mean imputation method, and a penalized EM algorithm incorporating non-random missingness (PEMM) [12].

## Results

In this manuscript we propose two new methods to handle qPCR non-detects that provide consistent estimates of the mean and variance of gene expression: MI and DirEst. MI does this by taking into account the uncertainty of the imputed data. The central idea of MI is to replace a set of missing data points with *M* sets of plausible values. These sets of values are independent between the imputations, but share a correlation structure within each complete data set. Unlike single imputation, this method captures the uncertainty in the imputed values. The resulting *M* complete data sets can be analyzed using standard statistical techniques, and the results can be combined and compared across the *M* datasets. Contradictory results may indicate that the imputed values drive the inference and not the observed data.

DirEst allows for direct estimation of within replicate means for each gene and sample type, and variances for each gene. This approach is applicable to the most common experimental designs and subsequent analyses, identification of genes that are differentially expressed between sample types. For this type of analysis, the within-replicate means and variances are sufficient statistics, meaning that all the information about the data log-likelihood is contained in these parameters [13]. A limitation of the DirEst approach is that individual expression values are unavailable, so analyses such as clustering or network modeling are not possible.

### A statistical model for qPCR non-detects

We propose the following generative model for qPCR data in which $Y_{ij}$ is the Cq value for gene *i* in sample *j*, some of which are missing (non-detects), $X_{ij}$ represents the fully observed Cq values, and $Z_{ij}$ indicates whether a Cq value was detected:

1 $$\begin{aligned} X_{ij}= & {} f(\theta _{ij}, \eta ) + \varepsilon _{ij} \nonumber \\ Y_{ij}= & {} {\left\{ \begin{array}{ll} X_{ij} &{} \text{ if } Z_{ij} = 1 \\ \text{ non-detect } &{} \text{ if } Z_{ij} = 0 \end{array}\right. } \nonumber \\ Pr(Z_{ij}=1)= & {} {\left\{ \begin{array}{ll} g(X_{ij}) &{} \text{ if } X_{ij} < S \\ 0 &{} \text{ if } \text{ otherwise }. \end{array}\right. } \end{aligned}$$

In this model, we assume that the fully observed Cq values, $X_{ij}$ are a function of the true gene expression, $\theta _{ij}$, non-biological factors, $\eta$, and random measurement error, $\varepsilon _{ij}$. The probability of a Cq value being detected is assumed to be a function of the Cq value itself, $g(X_{ij})$, for values below the detection limit, *S*. Note that the generative model proposed in [10] is a special case of the model described here. The logistic function is a natural choice for *g*() to describe the increasing probability of a Cq value being a non-detect; however, other sigmoidal functions would likely perform similarly. Here we focus on the following specific form of the relationship between $Z_{ij}$ and $X_{ij}$:

2 $$\begin{aligned} logit\Big (Pr(Z_{ij}=1) \Big )=\beta _0 + \beta _1 X_{ij}. \end{aligned}$$

Here, *β*_0_ and *β*_1_ describe the relationship between the probability of a non-detect and the potentially unobserved expression value $X_{{ij}}$. Specifically, $\beta _{0}$ is the log-odds of a non-detect when $X_{ij}=0$, and $\beta _{1}$ is the log odds ratio of a non-detect for a one unit increase in $X_{ij}$.

In practice, it is necessary to impose additional structure on the model described above based on the experimental design employed. While $f(\theta _{ij}, \eta )$ is flexible enough to capture more complex study designs, the vast majority of qPCR experiments seek to compare replicate samples from two or more sample-types. In this case, the model proposed above can be easily tailored to this type of experimental design. Specifically, we partition the samples ($j = 1, \dots , J$) into *K* sets of replicates, $J_k$, with $k(j) = k$ for $j\in J_k$. In Eq. 1, we simply replace $\theta _{ij}$ with $\theta _{ik(j)}$. Models of absolute and relative quantification are special cases of this model and are described in detail in the Methods.

### Simulation studies

To gain insights into the performance of the proposed MI and DirEst methodology, we constructed a simulation study (see Methods for details) to compare the bias and mean squared error (MSE) of the model parameter estimates from four imputation techniques: mean imputation, single imputation, multiple imputation, and direct estimation.

#### Performance assessments of the proposed methods

**Fig. 1:**
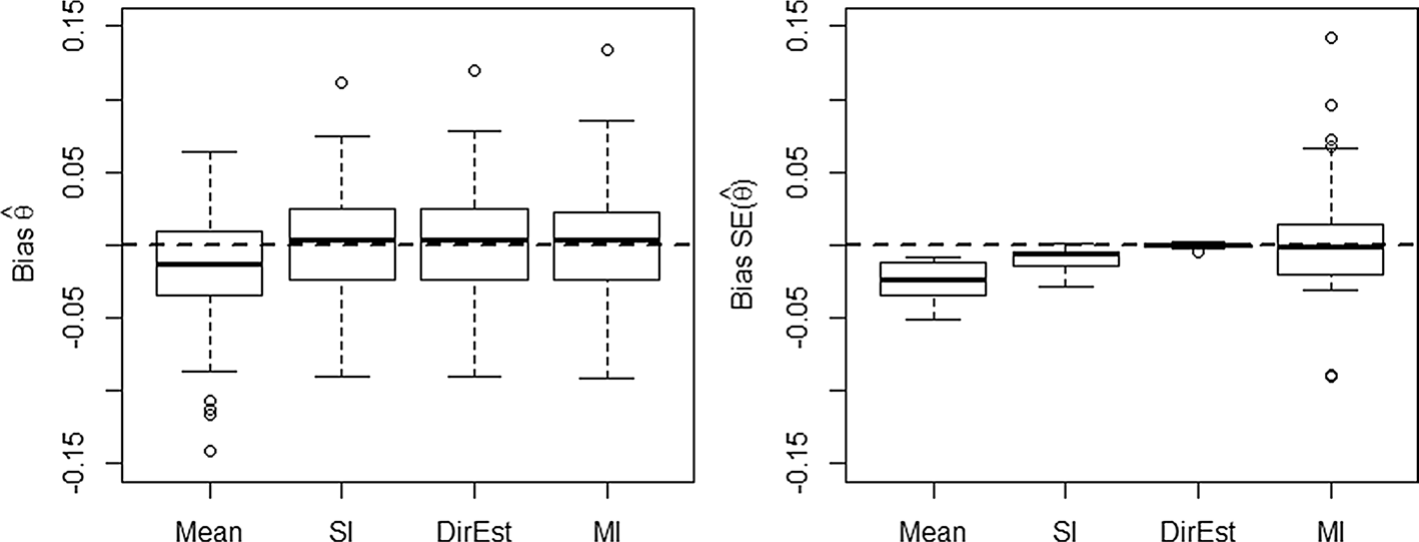
Comparison of methods to handle missing data based on 100 simulated data sets. The left and the right panels show the bias of ${\hat{\theta }}$ and the standard error of ${\hat{\theta }}$ respectively, calculated with mean imputation (Mean), single imputation (SI), direct estimation (DirEst), and multiple imputation (MI)

We expect the residual variance of gene expression to be underestimated when performing a SI procedure. To confirm this and assess the bias and MSE of ${\theta }_{ij(k)}$ and ${\sigma }^2_{\theta }$, we performed a simulation study with 16 genes. While the SI bias and MSE are small for both $\theta _{ij(k)}$ and $\sigma _{\theta }^2$, the SI bias for $\sigma _{\theta }^2$ is always negative (Fig. 1 and Additional file 1: Table 1). Note that for SI, ${\sigma }^2_{\theta } = {\sigma }^2_{i} / \#(J_k)$; therefore, we confirm that the SI procedure underestimates the residual variance.

Figure 1 displays boxplots of the bias for $\theta$ and ${\sigma }^2_\theta$ for all four methods. The mean imputation method underestimated both $\theta$ and $\sigma ^2_\theta$ in this study. These disadvantages of mean imputation are noticeable even for a relatively small proportion of missing values (5-10$\%$). Single imputation performed almost as well as multiple imputation and direct estimation with respect to the bias of ${\hat{\theta }}$; however, as expected SI underestimated ${\hat{\sigma }} ^2_\theta$. MI on average had similar performance to DirEst; however, the range of MI bias for ${\hat{\sigma }} ^2_\theta$ is generally wider than DirEst. In summary DirEst and MI produce the most accurate estimates of $\theta$ and $\sigma ^2_{\theta }$ in our assessments.

#### Direct estimation is robust to misspecification of the missing data mechanism

We used the same simulated data to assess the robustness of our method to model misspecification, in which the assumed functional form of *g*() is incorrect. We compared the performance of the method under three possible link functions: logit, probit, and cloglog. Each simulation used the model described in Eqs. 1 and 2. To assess the effect of the link function, we chose to fix the number of genes (*I*=16) and number of replicates (*m*=6) within each of the 6 sample types. In this case the correctly specified model is a logit link.

All three link functions yield very similar results in terms of bias and MSE for $\theta$ and $\sigma _{\theta }^2$ (Table 1). Logit link gives estimates closer to the true values followed by cloglog then probit. Typically researchers are interested in estimating average gene expression $\theta$ and its variability $\sigma _{\theta }^2$. All three link functions performed almost identically in estimating these parameters; therefore, the proposed method appears robust to the choice of link function in estimating gene expression means and variances in this simulation study. Because of the model robustness to the choice of the link function, in the remaining sections we present results using the logit link.

**Table 1:** Performance assessments of direct estimation and multiple imputation under misspecification of the missing data mechanism based on 100 simulated data sets with 16 genes and 6 replicates per dataset

	Direct estimation						Multiple imputation					
	Bias			MSE			Bias			MSE		
	Logit											
$\theta$	− 0.024	0.003	0.025	0.072	0.114	0.156	− 0.024	0.003	0.023	0.072	0.114	0.155
$\sigma ^2_\theta$	− 0.001	0.000	0.000	0.000	0.001	0.001	0.020	− 0.001	0.014	0.000	0.001	0.004
	Probit											
$\theta$	− 0.022	0.003	0.024	0.071	0.114	0.153	− 0.024	− 0.003	0.023	0.072	0.113	0.155
$\sigma ^2_\theta$	− 0.002	− 0.001	0.000	0.000	0.001	0.001	− 0.020	− 0.001	0.014	0.000	0.001	0.004
	Cloglog											
$\theta$	− 0.025	0.003	0.026	0.007	0.114	0.156	− 0.025	0.002	0.022	0.072	0.114	0.156
$\sigma ^2_\theta$	− 0.001	0.000	0.000	0.000	0.001	0.001	− 0.022	0.000	0.014	0.000	0.001	0.004

The 25th (left), 50th (center), and 75th (right) quantiles of the bias and mean squared error (MSE) are reported

#### The effect of sample size on performance

To assess the effect of sample size, we compared the performance on data sets with 16 genes and 4, 6, or 10 replicates for each sample type. An increase in the number of replicates has minimal to no effect on the accuracy of the parameters of interest, $\theta$ and $\sigma _{\theta }^2$ (Table 2). These results are consistent across both methods. Additionally, the MSEs of $\theta$ and $\sigma ^2_\theta$ stay consistent with an increase in the number of replicates. Similar results were obtained for DirEst for probit and cloglog links (Additional file 1: Tables 2 and 3 in Appendix B). We also compared the performance on data sets with 16 or 90 genes and different numbers of replicates (Additional file 1: Table 4 in Appendix B). Because the missing data mechanism is assumed to be shared across genes, an increased number of genes decreases the bias and MSE of ${\hat{\beta }}_0$ and ${\hat{\beta }}_1$; however, estimation of $\theta$ and $\sigma ^2$ is not effected.

**Table 2:** Performance assessments of direct estimation and multiple imputation for varying number of replicates based on 100 simulated data sets

	Direct estimation						Multiple imputation					
	Bias			MSE			Bias			MSE		
	k=4											
$\theta$	− 0.024	0.005	0.031	0.112	0.171	0.225	− 0.024	0.004	0.029	0.113	0.170	0.232
$\sigma ^2_\theta$	− 0.002	0.000	0.003	0.001	0.003	0.006	− 0.045	− 0.019	0.030	0.001	0.002	0.005
	k=6											
$\theta$	− 0.024	0.003	0.025	0.072	0.114	0.156	− 0.024	0.003	0.023	0.072	0.114	0.155
$\sigma ^2_\theta$	− 0.001	0.000	0.000	0.000	0.001	0.001	− 0.02	− 0.001	0.014	0.000	0.001	0.004
	k=10											
$\theta$	− 0.014	0.003	0.020	0.044	0.069	0.089	− 0.015	0.002	0.017	0.044	0.069	0.090
$\sigma ^2_\theta$	0.000	0.000	0.001	0.000	0.000	0.000	− 0.008	0.003	0.024	0.000	0.000	0.002

The 25th (left), 50th (center), and 75th (right) quantiles of the bias and MSE are reported

### Comparison of proposed methods using real data

We applied the proposed methodology to three experimentally-derived real datasets. The first dataset is composed of two cell types and three treatments [14] in which 1.84% of expression values are non-detects. The second dataset is a study of the effect of p53 and/or Ras mutations on gene expression [15] in which 2.77% of expression values are missing. The third dataset consists of nine gene perturbations with matched control samples [16] in which 1.24% of expression values are non-detects. As in the original publications, all three datasets were normalized to a reference gene, Becn1. Additional details regarding each of these datasets can be found in the original publications.

#### Difference in variance estimates between single imputation and direct estimation

We compared estimates of the variance from the DirEst procedure with SI estimates for absolute and relative quantification. The minimum, maximum, first, second and third quartiles for ${{\widehat{\sigma }}}^2_{MLE} - {\widehat{\sigma }}^2_{SI}$ are presented in Table 3 for all three datasets. The difference between the two variance estimates is usually small, but in Dataset 2 the variance estimates for the gene *Afp* differ by 35.38. In this dataset *Afp* has 13 non-detects out of 14 measurements. This is a concrete example of the difference in variance estimates increasing as the number of non-detects increases, in other words, the effect of a larger *Q* in Eq. 8.

**Table 3:** Summary statistics for the difference between estimates of within replicate variance: ${\widehat{\sigma }}^2_{MLE} - {\widehat{\sigma }}^2_{SI}$ in three real data sets

	Min.	1st. Qu.	Median	Mean	3rd. Qu.	Max.
Dataset 1	0.024	0.026	0.033	0.095	0.109	0.429
Dataset 2	0.006	0.009	0.027	1.014	0.110	35.380
Dataset 3	0.010	0.019	0.040	0.056	0.042	0.199

The individual differences in variance estimates for absolute and relative expression can be seen in Additional file 1: Figures 1 and 2 in Appendix C, respectively. Similar to the results shown in Table 3, most of the differences are fairly small with a few exceptions, especially in Dataset 2. However, even small differences may influence downstream analyses and lead to anti-conservative inference.

#### Performance assessment based on masked data

**Fig. 2:**
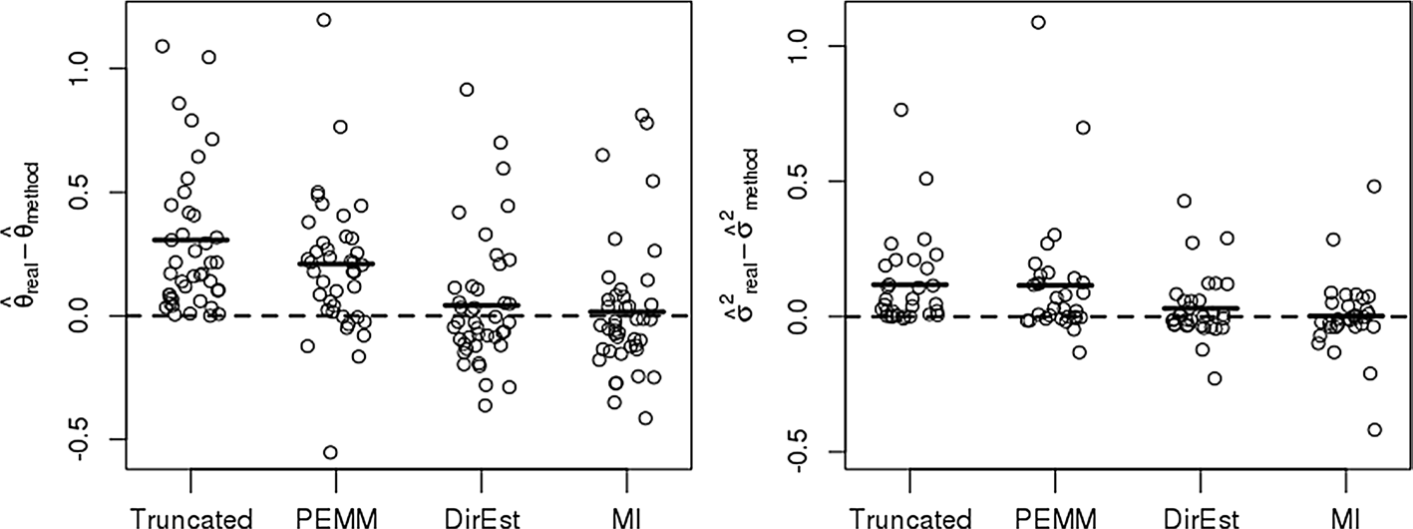
Comparison of methods for non-detects based on masked at Cq=30 values. The left panel shows the difference between $\theta$ estimated from the complete data and the estimate of $\theta$ obtained from the truncated data, applying PEMM, DirEst, and MI respectively. The right panel shows the difference between $\sigma ^2$ calculated from the complete data and four estimates of $\sigma ^2$: from the truncated data, PEMM, DirEst, and MI. Black solid lines represent means of the differences between parameter estimates from the complete data and parameter estimates from the masked data

As a part of the performance assessment we tested DirEst and MI on masked experimental data from [15]. The masking procedure is described in the Methods. We observe that both $\theta$ and $\sigma ^2$ are underestimated for all methods (Fig. 2). The largest differences between the true and estimated parameters are produced by the truncated data followed by PEMM. DirEst is an improvement over truncation and PEMM, and MI gives a slight improvement over DirEst for both parameters, $\theta$ and $\sigma ^2$. DirEst and MI on average provide estimates from the masked data that are close to the estimates obtained from the unmasked data. The estimated parameters of the missing data mechanism for DirEst and MI are $\hat{\beta }_0 = -879$ and ${\hat{\beta }}_1 = 29$, which accurately approximate the step function induced by the masking procedure.

#### Multiple imputation for absolute and relative quantification

We have implemented a MI procedure in the presence of non-detects for absolute quantification. To account for the uncertainty in the imputed values, we propose to use different variability sources and combinations thereof. The MI approach allows one to choose the number of complete data sets to impute for a set of non-detects, in contrast to SI which returns one complete data set of observed and imputed values. Moreover, we can study the impact of different sources of variation and the uncertainty introduced by the missing data. With DirEst we have some idea of the impact of non-detects, but MI allows us to see which of the sources has the largest impact on the imputed values and on the parameters of interest. In Additional file 1: Figure 3 in Appendix C, we show gene expression estimates produced by SI and MI for Datasets 1 and 2. MI can incorporate all sources of uncertainty at once (MI: all), pairs of sources (e.g. MI: $\theta ,\,\varepsilon$ or MI: fit, $\theta$), or one source at a time (MI: $\varepsilon$, MI: $\theta$, MI: fit).

In Additional file 1: Fig. 4 in Appendix C, we show the distribution of residuals for the observed data and the results of a SI procedure and several MI procedures for the missing data. Because a missing value likely represents slightly lower gene expression compared to the observed values from replicate samples, the majority of the residuals for the imputed values are slightly negative. While the medians are similar between the MI and SI results in both Datasets 1 and 2, the MI residuals often have a larger IQR because they incorporate additional sources of variability that are ignored by the SI procedure. The smallest impact on the distribution of the residuals is the uncertainty in the missing data mechanism, indicated as “MI: fit” in Additional file 1: Figures 3 and 4, followed by uncertainty in $\theta$. The biggest impact is measurement error, $\varepsilon$. Overall, the MI procedure better captures the uncertainty in estimates of absolute quantification.

Similar to absolute quantification, we have implemented an MI procedure in the presence of non-detects for relative quantification. While Datasets 1 and 2 focused on absolute expression, Dataset 3 focused on relative expression. The results for ten imputed data sets are presented in Additional file 1: Figure 5. The distribution of within replicate residuals in MI compared to SI appears to be very similar in the case where only uncertainty in $\theta$ is included in MI. Overall, the mean of the residual distribution stays relatively unchanged, but the IQR is wider due to the incorporated sources of variability in the model. In summary, the uncertainty in estimates of relative quantification are better captured by MI than by SI.

## Discussion

This paper has introduced two methods to account for missing data in qPCR experiments: MI and DirEst. Both methods treat qPCR non-detects as data missing not at random, and model the missing data mechanism with a two parameter sigmoidal curve. Using simulations, we showed that DirEst and MI can accurately estimate the first two moments of the distribution of gene expression and out perform SI and mean imputation. Additionally, we showed that the proposed methods are robust to model misspecification and perform well even with small sample sizes and without a large number of replicate samples.

A previous SI procedure, described in [10], preserves the first moment but introduces bias in the estimation of the variance. We have shown that this underestimation of the variability increases with the proportion of non-detects. Smaller estimated standard deviations lead to smaller p-values, which result in anti-conservative inference and a larger Type I error. This can lead to reporting significant results where there is no statistical difference.

We developed a MI procedure that incorporates different sources of uncertainty into the model. This approach is preferred when the actual expression estimates are required for analysis, for example in gene regulatory network modeling, clustering, or co-expression analysis. We also developed a method to estimate model parameters directly (DirEst). This method can be used when the mean and variance are sufficient statistics, e.g. when analyzing differences in average gene expression across groups. In addition to estimating absolute expression within each sample type, the methods we developed can be used to assess relative expression between sample-types and are applicable to any study design that can be expressed as a linear model.

In contrast to mean imputation or SI, both of the proposed methods avoid replacing missing data with a single value and subsequently analyzing the imputed values as if they are equally reliable as the observed values. DirEst avoids imputation entirely and instead directly estimates the model parameters, typically the within-group means and their associated standard errors. Our MI procedure accounts for the fact that some values are imputed by producing several sets of imputed values, which can be subsequently used to assess the effect of uncertainty in the imputation on downstream analyses.

In the proposed methods genes are treated as independent, and we assume a common residual variance across sample types for each gene, which is consistent with existing methodologies in genomics [17, 18]. However, some have advocated posing a dependence structure and using Bayesian shrinkage to estimate gene-specific variances [19]. Modeling the interdependence between genes is a potential source of further improvement to the proposed methods. Another current limitation is that the proposed methods require an observed value for a given gene in at least one replicate sample; however, it is possible for all the replicates of a given sample-type to be non-detects, in which case our methods are not applicable. Furthermore, it is important to distinguish between the lack of gene expression and the lack of detection of an expressed gene. This is particularly important when analyzing qPCR data near the limit of detection, which is common in both single-cell qPCR [20] and the analysis of circulating microRNAs [21].

A key assumption of the proposed methods is that a non-detect does not imply zero copies; instead, a small amount of initial copies coupled with the stochastic nature of PCR produces a non-zero probability of a non-detect. If this assumption does not hold, e.g. if some features are expected to be completely unexpressed in some samples, the average Cq value is no longer a sensible summary of the expression of those features within a sample type. In this case, one should consider alternatives, such as estimates of the proportion of samples in which a feature is expressed or the initial number of transcripts in each sample; however, the methods proposed in this manuscript are not directly applicable to such analyses. Finally, any method to handle missing data should be used with caution, its assumptions should be carefully examined, and sensitivity analyses should be performed to determine the extent to which the results depend on changes in these assumptions.

## Conclusions

Both MI and DirEst models are viable options for the analysis of qPCR data with non-detects for which the average Cq value is a reasonable summary statistic. These methods are implemented in the R/Bioconductor package, *nondetects*. Application of these methods will facilitate downstream analyses and increase confidence in the scientific conclusions drawn from qPCR experiments.

## Methods

### Model for absolute quantification

Absolute quantification is used to estimate the expression of a target transcript in one or more sample-types. For *absolute* gene expression we write the proposed model as follows:

3 $$\begin{aligned} X_{ij} = \theta _{ik(j)} + \delta _{j} + \varepsilon _{ij} \end{aligned}$$

where $X_{ij}$ are again the completely observed gene expression values, $\theta _{ik(j)}$ are the true values of gene expression for gene *i* in the sample-type *k* to which sample *j* belongs, $\delta _{j}$ represents a global shift in expression across samples, and $\varepsilon _{ij}$ now captures both biological and technical variability. In addition to estimating absolute expression within each sample-type, parameter estimates from this model can be used to assess relative expression between sample-types.

### Model for relative quantification

Relative quantification is used to estimate the change in expression of a target transcript between two sample types, typically experimental test and control samples. Due to the significant impact of batch effects on genomic data [22], it is increasingly common for experiments to include a matched control sample for each sample or group of samples analyzed. In this case, the parameters of interest are no longer the average expression within each sample type; rather, they are the differences in expression between the test and control samples. These control samples can be included in a model to directly adjust for batch effects. Specifically, we partition samples into $J^{'}_{n}$ batches, with $n(j) = n$ for $j\in J^{'}_{n}$, and introduce $\gamma _{in}$ into the model to capture the batch effect for gene *i* in samples from batch *n* as follows:

4 $$\begin{aligned} X_{ij}&= \Delta _{ik(j)}+ \gamma _{in(j)} + \delta _{j} + \varepsilon _{ij}, \nonumber \\ \Delta _{ik(j)}&= \theta _{ik(j)} - \theta ^{Control}_{ij} \end{aligned}$$

Here, the parameter of interest, $\Delta _{ik(j)}$, is the difference in expression between the test and control sample. We assume that control samples are denoted by $k=0$ and $\Delta _{i0}=0~\forall i$, such that:

5 $$\begin{aligned} X_{ij} = {\left\{ \begin{array}{ll} \Delta _{ik(j)} + \gamma _{in(j)} + \delta _{j} + \varepsilon _{ij} &{} \text{ if } j \notin J_0 \\ \gamma _{in(j)} + \delta _{j} + \varepsilon _{ij} &{} \text{ if } j \in J_0 \end{array}\right. } \end{aligned}$$

Rarely, non-detects may occur in the control samples as well as in the test samples. In this case we propose to use a two step process: first, apply the model in Eq. 3 for the control samples and perform SI, MI, or DirEst; second, use the model in Eq. 4, to obtain the estimates of the non-detects for the test samples.

### Estimation of model parameters

We perform parameter estimation by using an Expectation Conditional Maximization (ECM) procedure [23], in this section we outline this process. Our algorithm is given in more detail in Additional file 1: Appendix D. Let $Y=(X,W)$ be the observed data, where *X* represents the complete data and *W* denotes the non-detects. We first replace the non-detects with initial estimates and obtain an initial estimate of the missing data mechanism, $(\beta _0^{(0)}, \beta _1^{(0)})$. In the first step of the ECM algorithm, we calculate $E^{(t)}(W)$ and update $\theta _{ij}^{(t)}$, and in the second step, based on the results of the first step, we calculate $E^{(t)} (W^2)$ and $\sigma _i^{2^{(t)}}$. This process is repeated until the change in the likelihood is less than a specified threshold.

### Improved estimation of the missing data mechanism

It is possible to observe perfect separation between observed and non-detected transcripts. This is a common problem in regression with binary predictors. Prediction of the parameter values in this case becomes unstable; however, one can use a Bayesian procedure to obtain stable estimates of the generalized linear regression coefficients [24]. We adopt this approach when estimating the parameters of the missing data mechanism, $(\beta _0, \beta _1)$.

### Multiple imputation

We incorporated different sources of variability in the MI procedure: non-systematic variation, uncertainty in the linear model parameters ($\theta$ in Eqs. 3 and 4), and parameters of the missing data mechanism ($\beta _0$ and $\beta _1$ in Eq. 2), as well as each combination of these sources of variability.

#### Uncertainty in linear model parameters

When the data has missing values, the estimates of the model parameters contain an additional amount of uncertainty due to the missing data. We can account for this added uncertainty by introducing additional variation in the parameter estimates. Instead of using point estimates ${\hat{\theta }}$, we draw *M* different ${\hat{\theta }}_m$ $(m=1, \ldots , M)$ from the estimated distribution of $\theta \sim {\mathrm {N}}({\hat{\mu }}_\theta , {\hat{\sigma }}_\theta )$.

#### Uncertainty in the missing data mechanism

Similarly, one can account for the uncertainty in the missing data mechanism by introducing additional variability in the corresponding parameter estimates. To preserve the dependence between the parameters, we assume $(\beta _0,\beta _1)$ are jointly ${\mathrm {MVN}}({\hat{\mu }}_\beta , {\hat{\Sigma }}_\beta )$. Researchers can draw *M* pairs of $(\beta _0,\beta _1)$ from the estimated distribution and use these estimates in the imputation procedure. This step introduces additional variability in the resulting complete data sets that reflects uncertainty in the estimated logistic regression model parameters.

#### Biological variability and measurement error

Suppose the model parameters are known and we are interested in applying MI to obtain an estimate of differential gene expression. In this case, given all the model parameters, the imputed values will be identical and equal to the conditional expectation of the missing data point without additional variability. Such a result is undesirable, as it will lead to artificially small variance estimates. To better estimate the uncertainty of the missing value itself under known true parameters of the modeling framework, we must include non-systematic biological variability and measurement error in the MI procedure. We assume that these sources of variability together are normally distributed with mean zero and variance equal to the residual variance from the EM procedure.

### Direct estimation of model parameters

An alternative approach to handling missing data is to directly estimate the parameters of interest. For a fixed gene, in sample *j*, let $w_j$ denote an unobserved value of the gene expression $y_j$ and let $z_j$ be an indicator of an observed expression value as in Eq. 1. The MLE of the variance for a given gene is:

6 $$\begin{aligned} {\widehat{\sigma }}^2_{MLE}&= \frac{1}{J} \sum _{j=1}^J\Big ((E(w_j^2)-2E(w_j)\theta _{k(j)} +\theta _{k(j)}^2)(1-z_j) \nonumber \\&+ (y_j^2-2 y_j\theta _{k(j)} +\theta _{k(j)}^2 )z_j \Big ). \end{aligned}$$

In contrast, the sample variance estimate following SI for a given gene is:

7 $$\begin{aligned} {\widehat{\sigma }}^2_{SI}&= \frac{1}{J} \sum _{j=1}^J \Big ((E(w_j)^2-2E(w_j)\theta _{k(j)} +\theta _{k(j)}^2 )(1-z_j) \nonumber \\&\quad + (y_j^2-2 y_j\theta _{k(j)} +\theta _{k(j)}^2 )z_j \Big ). \end{aligned}$$

Derivations of these equations are given in Appendix A.

These equations differ exclusively in the first element within the summation: $E(w_j^2)$ versus $E(w_j)^2$. Taking the difference between Eqs. (6) and (7) yields:

8 $$\begin{aligned} {\widehat{\sigma }}^2_{MLE} - {\widehat{\sigma }}^2_{SI}&= \frac{1}{J} \sum _{j=1}^J \Big ((E(w_j^2) - (E(w_j)^2)(1-z_j) \Big ) \nonumber \\&= \frac{1}{J} \sum _{j \in J, z_j=0} \Big (E(w_j^2) - (E(w_j)^2 \Big ) = Q \sigma _{w} \propto \sigma _{w} > 0, \end{aligned}$$

where $Q >0$ is the proportion of non-detects for the given gene. Since $E(w_j^2)$ is greater then $E(w_j)^2$, the variance estimated after SI is smaller than the MLE of the variance. As the number of non-detects increases, *Q* increases, and hence the difference between ${{\hat{\sigma }}}^2_{MLE}$ and ${{\hat{\sigma }}}^2_{SI}$ increases with the number of non-detected values. Note that these relationships hold for any given gene.

### Simulation study design

We performed simulation studies with either 16 or 90 genes, 6 sample-types, and 4, 6, or 10 replicates within each sample-type, such that $J_k$ is the same size $\forall \, k$. We denote the number of replicates within each sample type as *m*. Finally, we assume a common missing data mechanism parameterized as in Eq. 2. The values of parameters in the simulations were chosen at levels close to the estimated values from [14]. Specifically, we set $\beta _0=-35.7$ and $\beta _1=1$; the $\sigma ^2_i$ were generated from *Unif*(0.06, 1.3); $\theta _{ij(k)}$ were generated from $N(\mu _\theta , \sigma ^2_{\theta } I)$, where $\sigma ^2_{\theta }=3$; $\mu _{\theta }$ was generated from a truncated normal distribution with mean 31, standard deviation of 3.5 and the truncation range from 20 to 40.5. We set $\delta _j$ to 0, simulated $\varepsilon$ from $N(0, \sigma ^2_{i} I)$, and simulated the complete data by combining $\theta$, $\delta$ and $\varepsilon$ as in Eq. 3. To obtain the missing data indicators, we drew from a $Binom(p_{ij(k)})$, where $p_{ij(k)}=P(Z_{ij}=1)$ is calculated as in Eq. 2 for each data point. The individual generated data points we replaced with 40 according to missing data indicators or if the Ct value was greater or equal to 40. We compared performance under logit, probit and cloglog links. For each scenario we repeated this procedure 100 times. To summarize the performance of each estimation technique, we report the 25th, 50th, and 75th quantiles of each assessment measure for all genes and samples. For example, in the case of 16 genes and 6 sample-types, there are 16 different $\sigma ^2_{i}$ and $16 \times 6$ = 96 distinct values of $\theta _{ij(k)}$ for each simulated data set.

### A penalized EM algorithm incorporating non-random missingness (PEMM)

We compared the proposed DirEst and MI methods to the PEMM algorithm proposed by [12]. PEMM incorporates an Inverse-Wishart penalty into an EM algorithm to estimate the mean and covariance of multivariate Normal data in the presence of missing values. PEMM was initially applied to proteomics abundance levels under the following assumptions: (1) the probability of a missing value does not depend on the missingness of other values given the abundance data and other covariates and (2) the missingness of each feature is independent of the abundance of other features and covariates. In contrast in our methods we assume one common missing data mechanism and estimate it based on all the information available. While PEMM was developed for proteomics data, we have adapted it to qPCR data by transforming the Ct values to a scale similar to proteomic abundance data by subtracting each Ct value from the largest possible value. PEMM proposing the following model of the missing data mechanism: $Pr(Y|Z=1) = c \times \exp ^{(-\phi \times Y)}$ for positive abundance *Y*, constant *c*, and tuning parameter $\phi$. We tested PEMM with different values of the parameter in the missing-data mechanism $\phi =0.5, \, 1, \,2$, and chose the value $\phi =1$ that gave the highest correlation between complete data mean estimates and PEMM mean estimates. The PEMM algorithm is implemented in the R package PEMM (version 1.0).

### Masking procedure for the experimental data to assess the methods performance

To assess the applicability of the proposed methods to real data, we used a masking procedure to create missing data for which the observe values are known. First, all the genes with missing data were removed, resulting in a completely observed data set from which we obtained ${{\hat{\theta }}}_{complete}$ and ${{\hat{\sigma }}}^2_{complete}$. Second, the new detection limit was set at Ct=30, and expression values greater than 30 were masked. Importantly, rather than creating missing points based on the probabilistic model, which would require assumptions regarding the unknown missing data mechanism, we truncated the data. Given that PEMM makes different assumptions about the missing data mechanism than DirEst and MI, we restrict our comparison between these methods to the masked data. We calculated ${{\hat{\theta }}}_{method}$ and ${\hat{\sigma }}^2_{method}$ for truncated at 30 data, PEMM, DirEst, and MI, and compared them to the estimates from the complete data.

### Supplementary information

**Additional file 1.** Supplementary Materials: The supplementary materials contain derivations of the variance estimates for SI and MLE and their difference in Appendix A. In Appendix B we present additional simulation results. Supplementary Figures are shown in Appendix C.

## Data Availability

All experimental data used in this work is available in the R/Bioconductor package *nondetects*. R scripts and data to reproduce all figures and tables are available at: https://doi.org/10.5281/zenodo.2554418

## Abbreviations

cDNA: complementary deoxyribonucleic acid
Cq: quantification cycle
DirEst: direct estimation
DNA: deoxyribonucleic acid
ECM: expectation conditional maximization
EM: expectation maximization
IQR: interquartile range
MI: multiple imputation
MLE: maximum likelihood estimate
mRNA: messenger ribonucleic acid
MSE: mean squared error
PCR: polymerase chain reaction
PEMM: penalized expectation maximization incorporating non-random missingness
qPCR: quantitative real-time polymerase chain reaction
SI: single imputation

## References

[1] Mullis KB, Erlich HA, Arnheim N, Horn GT, Saiki RK, Scharf SJ. Process for amplifying, detecting, and/or-cloning nucleic acid sequences. Google Patents. US Patent 4,683,195. 1987.

[2] Bartlett JM, Stirling D. A short history of the polymerase chain reaction. PCR protocols. 2003;3–6. 10.1385/1-59259-384-4:3 12958470

[3] Lefever S, Hellemans J, Pattyn F, Przybylski D, Taylor C, Geurts R, Untergasser A, Vandesompele J, Consortium R (2009). Rdml: structured language and reporting guidelines for real-time quantitative pcr data. Nucleic Acids Res.

[4] Morrison TB, Weis JJ, Wittwer CT (1998). Quantification of low-copy transcripts by continuous sybr green i monitoring during amplification. Biotechniques.

[5] Pfaffl M. Development and validation of an externally standardised quantitative insulin-like growth factor-1 rt-pcr using lightcycler sybr green i technology. In: Rapid Cycle Real-Time PCR. Springer; 2001. p. 281–91.

[6] Pfaffl MW (2001). A new mathematical model for relative quantification in real-time rt-pcr. Nucleic Acids Res.

[7] Rutledge R (2004). Sigmoidal curve-fitting redefines quantitative real-time pcr with the prospective of developing automated high-throughput applications. Nucleic Acids Res.

[8] Spiess A-N, Feig C, Ritz C (2008). Highly accurate sigmoidal fitting of real-time pcr data by introducing a parameter for asymmetry. BMC Bioinform.

[9] Gelman A, Hill J (2006). Data analysis using regression and multilevel/hierarchical models.

[10] McCall MN, McMurray HR, Land H, Almudevar A. On non-detects in qpcr data. Bioinformatics. 2014;30(16):2310–6. 10.1093/bioinformatics/btu239http://bioinformatics.oxfordjournals.org/content/30/16/2310.full.pdf+html. 10.1093/bioinformatics/btu239 PMC4133581 24764462

[11] Komisarof J, McCall M, Newman L, Bshara W, MohlerJL Morrison C, Land H (2017). A four gene signature predictive of recurrent prostate cancer. Oncotarget.

[12] Chen LS, Prentice RL, Wang P (2014). A penalized em algorithm incorporating missing data mechanism for gaussian parameter estimation. Biometrics.

[13] Fisher RA (1922). On the mathematical foundations of theoretical statistics. Philos Trans R Soc Lond Ser A Contain Pap Math Phys Character.

[14] Sampson ER, McMurray HR, Hassane DC, Newman L, Salzman P, Jordan CT, Land H (2013). Gene signature critical to cancer phenotype as a paradigm for anticancer drug discovery. Oncogene.

[15] McMurray HR, Sampson ER, Compitello G, Kinsey C, Newman L, Smith B, Chen S-R, Klebanov L, Salzman P, Yakovlev A (2008). Synergistic response to oncogenic mutations defines gene class critical to cancer phenotype. Nature.

[16] Almudevar A, McCall MN, McMurray H, Land H (2011). Fitting boolean networks from steady state perturbation data. Stat Appl Genet Mol Biol.

[17] Anders S, Huber W (2010). Differential expression analysis for sequence count data. Genome Biol.

[18] Robinson MD, McCarthy DJ, Smyth GK (2010). edger: a bioconductor package for differential expression analysis of digital gene expression data. Bioinformatics.

[19] Ritchie ME, Phipson B, Wu D, Hu Y, Law CW, Shi W, Smyth GK (2015). limma powers differential expression analyses for rna-sequencing and microarray studies. Nucleic Acids Res.

[20] McDavid A, Finak G, Chattopadyay PK, Dominguez M, Lamoreaux L, Ma SS, Roederer M, Gottardo R (2012). Data exploration, quality control and testing in single-cell qpcr-based gene expression experiments. Bioinformatics.

[21] De Ronde MW, Ruijter JM, Lanfear D, Bayes-Genis A, Kok MG, Creemers EE, Pinto YM, Pinto-Sietsma S-J (2017). Practical data handling pipeline improves performance of qpcr-based circulating mirna measurements. RNA.

[22] Leek JT, Scharpf RB, Bravo HC, Simcha D, Langmead B, Johnson WE, Geman D, Baggerly K, Irizarry RA (2010). Tackling the widespread and critical impact of batch effects in high-throughput data. Nat Rev Genet.

[23] Meng X-L, Rubin DB (1993). Maximum likelihood estimation via the ECM algorithm: a general framework. Biometrika.

[24] Gelman A, Jakulin A, Pittau MG, Su Y-S (2008). A weakly informative default prior distribution for logistic and other regression models. Ann Appl Stat.

