# Correction: Multiple imputation and direct estimation for qPCR data with non-detects

**DOI:** 10.1186/s12859-024-05653-5

**Published:** 2024-02-07

**Authors:** Valeriia Sherina, Helene R. McMurray, Winslow Powers, Harmut Land, Tanzy M. T. Love, Matthew N. McCall

**Affiliations:** 1 Department of Biostatistics and Computational Biology, University of Rochester Medical Center, 265 Crittenden Blvd., Rochester, NY 14642 USA; 2 Department of Biomedical Genetics, University of Rochester Medical Center, 601 Elmwood Ave, Rochester, NY 14642 USA; 3 Department of Pathology and Laboratory Medicine, University of Rochester Medical Center, 601 Elmwood Ave., Rochester, NY 14642 USA; 4 Department of Biomedical Engineering, University of Rochester, 201 Robert B. Goergen Hall, Rochester, NY 14627 USA

**Correction: BMC Bioinformatics (2020) 21:545** 10.1186/s12859-020-03807-9

Following the publication of the original article [1], the authors identified that Appendix D was missing in Additional file 1. The additional file has been updated.

The original article [1] has been corrected.

### Supplementary Information

**Additional file 1. Supplementary Materials**: The supplementary materials contain derivations of the variance estimates for SI and MLE and their difference in Appendix A. In Appendix B we present additional simulation results. Supplementary Figures are shown in Appendix C. Appendix D Estimation via Expectation Conditional Maximization (ECM).
