# Supplementary material for: Multiple imputation and direct estimation for qPCR data with non-detects
Source: BMC Bioinformatics. 2020 Nov 26;21:545. doi: 10.1186/s12859-020-03807-9 (PMC7693525; doi:10.1186/s12859-020-03807-9)
Supplement: Supplementary file 1 — Additional file 1. Supplementary Materials: The supplementary materials contain derivations of the variance estimates for SI and MLE and their difference in Appendix A. In Appendix B we present additional simulation results. Supplementary Figures are shown in Appendix C. [file 12859_2020_3807_MOESM1_ESM.pdf]

Supplementary Materials for Multiple imputation and direct estimation for qPCR data with non-detects

Supplementary materials contain derivations of the variance estimates for SI and MLE and their difference in Appendix A. In Appendix B we present tables with the simulation results. Supplementary Figures are shown in Appendix C. Appendix D describes the Expectation Conditional Maximization (ECM) procedure.

Appendix A - Difference in variance estimates

Assume Ct values follow a normal distribution, denoted by $y|X$, where $X\beta$ represents the gene and sample-type specific mean and σ^2 is a common variance:

$$y|X \sim N(X\beta, \sigma^2 I).$$

For a fixed gene i, y is (J x 1), X is (J x K), β is (K x 1). The likelihood can be written:

$$\begin{aligned} L(\beta,\sigma^2|X) &= -\frac{J}{2}log(2\pi) - \frac{J}{2}log(\sigma^2) - \frac{1}{2\sigma^2}(y - X\beta)'(y - X\beta) \\ &= -\frac{J}{2}log(2\pi) - \frac{J}{2}log(\sigma^2) - \frac{1}{2\sigma^2}\sum_{j=1}^{J}(y_j - \sum_{k=1}^{K} x_{jk}\beta_k)^2 \end{aligned}$$

Let $z_j = 1$ if y_j observed, and $z_j = 0$ if y_j is missing, and denote missing y_j by w_j.

$$\begin{aligned} L(\beta,\sigma^2|X) &= -\frac{J}{2}log(2\pi) - \frac{J}{2}log(\sigma^2) - \frac{1}{2\sigma^2}[\sum_{j=1}^{J}(y_j - \sum_{k=1}^{K} x_{jk}\beta_k)^2 z_j \\ &+ \sum_{k=1}^{K}(w_j - \sum_{k=1}^{K} x_{jk}\beta_k)^2)(1 - z_j)] \end{aligned}$$

$$\begin{aligned} E[L(\beta,\sigma^2|X)] &\propto -\frac{J}{2}log(\sigma^2) - \frac{1}{2\sigma^2}[\sum_{j=1}^{J}(y_j - \sum_{k=1}^{K} x_{jk}\beta_k)^2 z_j \\ &+ \sum_{j=1}^{J} E(w_j - \sum_{k=1}^{K} x_{jk}\beta_k)^2(1 - z_j) \\ &+ \sum_{j=1}^{J}(E(w_j^2) - 2E(w_j)\sum_{k=1}^{K} x_{jk}\beta_k + (\sum_{k=1}^{K} x_{jk}\beta_k)^2)(1 - z_j)] \end{aligned}$$

Let $\theta_{k(j)} = \sum_{k=1}^{K} x_{jk}\beta_k$

$$
\begin{aligned}
E[L(\theta, \sigma^2|X)] &\propto -\frac{J}{2}log(\sigma^2) - \frac{1}{2\sigma^2}[\sum_{j=1}^{J}(y_j - \theta_{k(j)})^2 z_j \\
&+ \sum_{j=1}^{J}(E(w_j^2) - 2E(w_j)\theta_{k(j)} + \theta_{k(j)}^2)(1 - z_j)] \\
&\propto -\frac{J}{2}log(\sigma^2) - \frac{1}{2\sigma^2}\sum_{j=1}^{J}(y_j^2 z_j + E(w_j^2)(1 - z_j)) \\
&- \frac{1}{2\sigma^2}\sum_{j=1}^{J}[(-2y_j\theta_{k(j)} + z_j\theta_{k(j)}^2) + (-2E(w_j)\theta_{k(j)} + \theta_{k(j)}^2)(1 - z_j)]
\end{aligned}
$$

Let $U_j = (y_j, E(w_j))$,

$$
\underset{\theta}{argmax}\, E[L(\theta, \sigma^2|X)] = \underset{\theta}{argmax}\, [-\frac{1}{2\sigma^2}\sum_{j=1}^{J}[(-2\theta_{k(j)}U_j + \theta_{k(j)}^2)]]
$$

$$
\begin{aligned}
E[L(\theta, \sigma^2|X)] &\propto -\frac{J}{2}log(\sigma^2) - \frac{1}{2\sigma^2}[\sum_{j=1}^{J}(y_j - \theta_{k(j)})^2 z_j + \sum_{j=1}^{J}(E(w_j^2) - 2E(w_j)\theta_{k(j)} + \theta_{k(j)}^2)(1 - z_j)] \\
&\propto -\frac{J}{2}log(\sigma^2) - \frac{1}{2\sigma^2}\sum_{j=1}^{J}(y_j^2 z_j + E(w_j^2)(1 - z_j)) \\
&- \frac{1}{2\sigma^2}\sum_{j=1}^{J}[(-2y_j\theta_{k(j)} + \theta_{k(j)}^2)z_j + (-2E(w_j)\theta_{k(j)} + \theta_{k(j)}^2)(1 - z_j)]
\end{aligned}
$$

Let $U_j = (y_j, E(w_j))$,

$$
\underset{\theta}{argmax}\, E[L(\theta, \sigma^2|X)] = \underset{\theta}{argmax}\, [-\frac{1}{2\sigma^2}\sum_{j=1}^{J}[(-2\theta_{k(j)}U_j + \theta_{k(j)}^2)]]
$$

One can update θ treating $E(w_j)$ as "data", then update σ^2 as:

$$
\frac{1}{J}[\sum_{j=1}^{J}(y_j - \theta_{k(j)})^2 z_j + \sum_{j=1}^{J}(E(w_j^2) - 2\theta_{k(j)}E(w_j) + \theta_{k(j)}^2)(1 - z_j)].
$$

Let $\sum_{j=1}^{J}\Big((E(w_j^2) - 2E(w_j)\theta_{k(j)} + \theta_{k(j)}^2)(1 - z_j) + (y_j^2 - 2y_j\theta_{k(j)} + \theta_{k(j)}^2)z_j\Big) = S$.

Then,

$$
\underset{\sigma^2}{argmax}\, E[L(\theta, \sigma^2|X)] = \underset{\sigma^2}{argmax}\big(-\frac{J}{2}log(\sigma^2) - \frac{1}{2\sigma^2}S\big).
$$

The MLE of the variance for any gene is:

$$
\widehat{\sigma}_{MLE}^2 = \frac{1}{J}\sum_{j=1}^{J}\Big((E(w_j^2) - 2E(w_j)\theta_{k(j)} + \theta_{k(j)}^2)(1 - z_j) + (y_j^2 - 2y_j\theta_{k(j)} + \theta_{k(j)}^2)z_j\Big).
$$

Appendix B - Additional Simulation results

Supplementary Table 1: Direct estimation, probit link. The 25th (small left), 50th (large center), and 75th (small right) quantiles of the bias and MSE are reported.

	Bias			MSE		
16 genes	k=4					
β_0		15.069			256.786	
β_1		-0.425			0.204	
θ	-0.022	0.007	0.033	0.112	0.172	0.229
σ^2	-0.008	-0.004	0.016	0.016	0.050	0.108
16 genes	k=6					
β_0		16.31			276.39	
β_1		-0.459			0.219	
θ	-0.022	0.003	0.024	0.071	0.114	0.153
σ^2	-0.009	-0.003	0.003	0.012	0.029	0.048
16 genes	k=10					
β_0		15.499			250.222	
β_1		-0.435			0.198	
θ	-0.014	0.003	0.017	0.044	0.069	0.089
σ^2	-0.003	0.005	0.008	0.006	0.019	0.031
90 genes	k=4					
β_0		1.019			7.896	
β_1		-0.034			0.007	
θ	-0.022	0.009	0.045	0.121	0.172	0.253
σ^2	-0.018	-0.004	0.009	0.025	0.045	0.098
90 genes	k=6					
β_0		-0.107			5.427	
β_1		0.000			0.004	
θ	-0.016	0.005	0.034	0.083	0.113	0.16
σ^2	-0.015	-0.003	0.007	0.016	0.029	0.053
90 genes	k=10					
β_0		-1.076			4.077	
β_1		0.029			0.003	
θ	-0.012	0.005	0.023	0.049	0.069	0.096
σ^2	-0.011	-0.003	0.006	0.009	0.016	0.03

Supplementary Table 2: Direct estimation, cloglog link. The 25th (small left), 50th (large center), and 75th (small right) quantiles of the bias and MSE are reported.

	Bias			MSE		
16 genes	k=4					
β_0		6.089			70.003	
β_1		-0.189			0.062	
θ	-0.024	0.005	0.029	0.111	0.168	0.225
σ^2	-0.012	-0.005	0.01	0.019	0.043	0.09
16 genes	k=6					
β_0		7.509			71.845	
β_1		-0.228			0.065	
θ	-0.025	0.003	0.026	0.071	0.114	0.156
σ^2	-0.006	-0.003	0.003	0.012	0.029	0.048
16 genes	k=10					
β_0		6.72			59.014	
β_1		-0.206			0.054	
θ	-0.016	0.002	0.018	0.044	0.069	0.09
σ^2	-0.002	0.004	0.011	0.006	0.019	0.03
90 genes	k=4					
β_0		8.754			80.535	
β_1		-0.266			0.074	
θ	-0.024	0.009	0.042	0.121	0.172	0.253
σ^2	-0.018	-0.004	0.008	0.025	0.045	0.098
90 genes	k=6					
β_0		-0.091			5.426	
β_1		-0.001			0.004	
θ	-0.016	0.005	0.034	0.083	0.113	0.160
σ^2	-0.015	-0.003	0.007	0.016	0.029	0.053
90 genes	k=10					
β_0		-1.065			4.053	
β_1		0.028			0.003	
θ	-0.012	0.005	0.024	0.049	0.069	0.096
σ^2	-0.011	-0.003	0.006	0.009	0.016	0.03

Supplementary Table 3: Mean imputation results, logit link. The 25th (small left), 50th (large center), and 75th (small right) quantiles of the bias and MSE are reported.

	Bias			MSE		
16 genes	k=4					
θ	-0.039	-0.005	0.019	0.105	0.173	0.225
σ^2	-0.258	-0.198	-0.098	0.021	0.063	0.103
16 genes	k=6					
θ	-0.035	-0.013	0.009	0.071	0.114	0.155
σ^2	-0.194	-0.143	-0.073	0.012	0.040	0.067
16 genes	k=10					
θ	-0.035	-0.009	0.008	0.044	0.069	0.090
σ^2	-0.145	-0.100	-0.051	0.006	0.024	0.040

Appendix C - Supplementary Figures

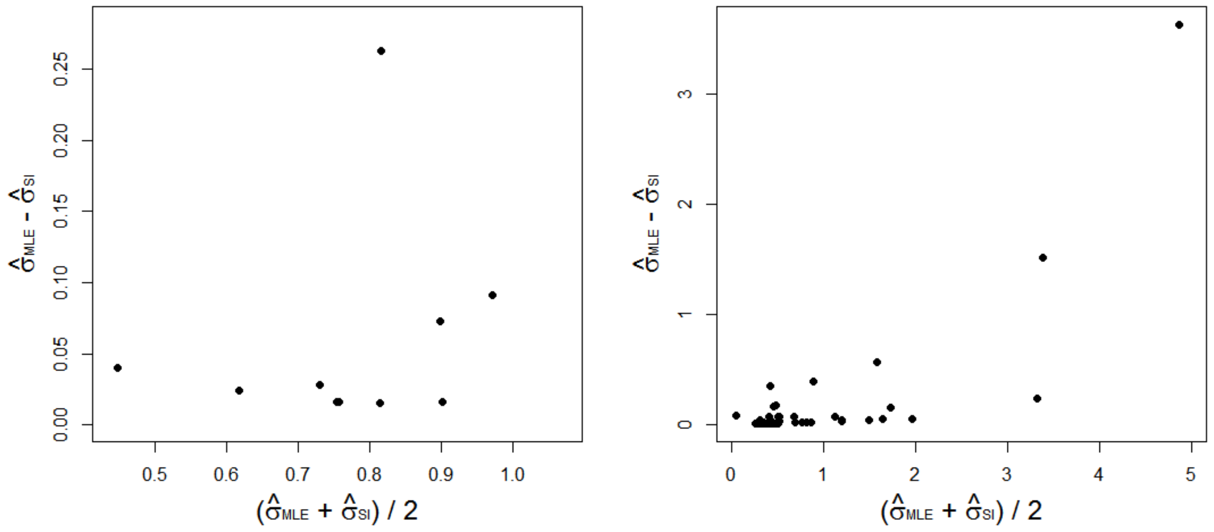

Supplementary Figure 1: The difference in gene variability between SI and DirEst. The difference in the standard deviation estimates for the two data sets: Dataset 1 on the left and Dataset 2 on the right. In the labels, $\hat{\sigma}_{MLE}$ - the direct Maximum Likelihood Estimate of σ; $\hat{\sigma}_{SI}$ - the estimate of σ from SI. On the horizontal axis is the average of the two estimates of the standard deviation, and the difference between the direct MLE estimate and the SI estimate of σ is on the vertical axis. The genes without missing values are not presented on the plot, because SI and MLE standard deviation estimates are identical. As noted in Section 2.3 of the manuscript, the variance of the gene expression is being underestimated by SI in comparison to the direct MLE estimate of the variability and more substantially underestimated when the variance is large.

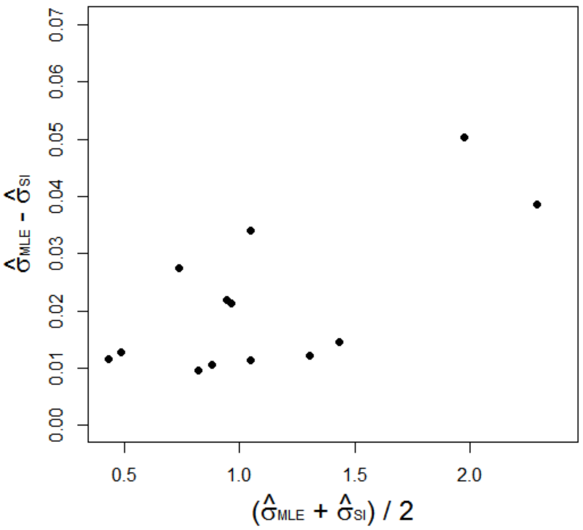

Supplementary Figure 2: The difference in the variance estimates for the Dataset 3. Similar to Figure 1, on the horizontal axis is the average of the two estimates of the standard deviation, and the difference between the direct MLE estimate and the SI estimate of σ is on the vertical axis.

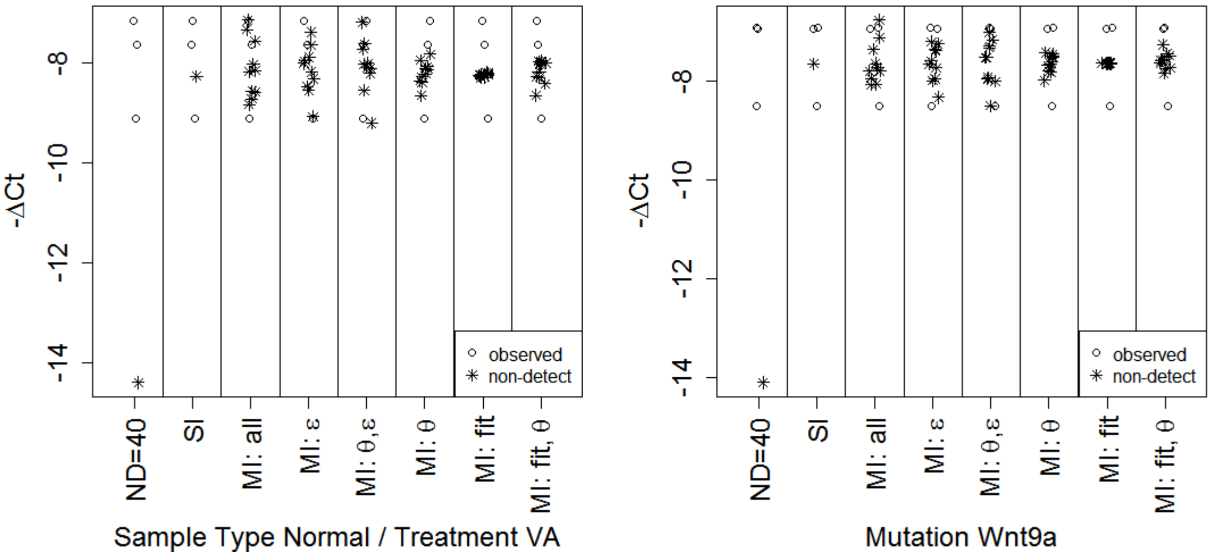

Supplementary Figure 3: Examples of differential gene expression produced by replacing non-detects with values of 40, applying SI, and different combinations of MI. The left panel shows the expression of Gpr149 in the combination of Normal sample type with VA treatment from Dataset 1. The panel on the right shows the response of Pdlim2 to Ras mutation from Dataset 2. In each picture ΔCt values produced by replacing a non-detect with imputed values are shown as asterisks.

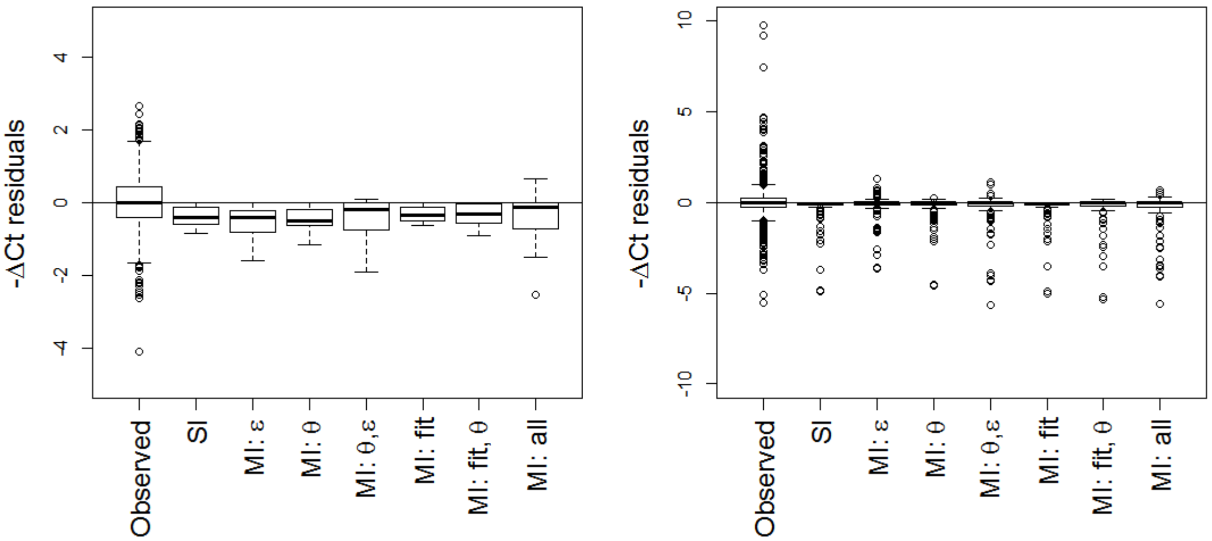

Supplementary Figure 4: Within replicate residuals stratified by the presence and handling of non-detects. The average ΔCt values were calculated over the replicates for gene i and sample-type k. The residuals, for each gene and sample-type were summarized and are plotted here. Both panels show the distribution of residuals from left to right: without non-detects in dataset, with SI applied to the data, with the application of MI with different sources of variability (noise, liner model parameters - θ, combination of the noise and θ, parameters of the logistic regression model - β's respectively), combination of uncertainty in θ and β's, and the combination of the three sources of variability combined.

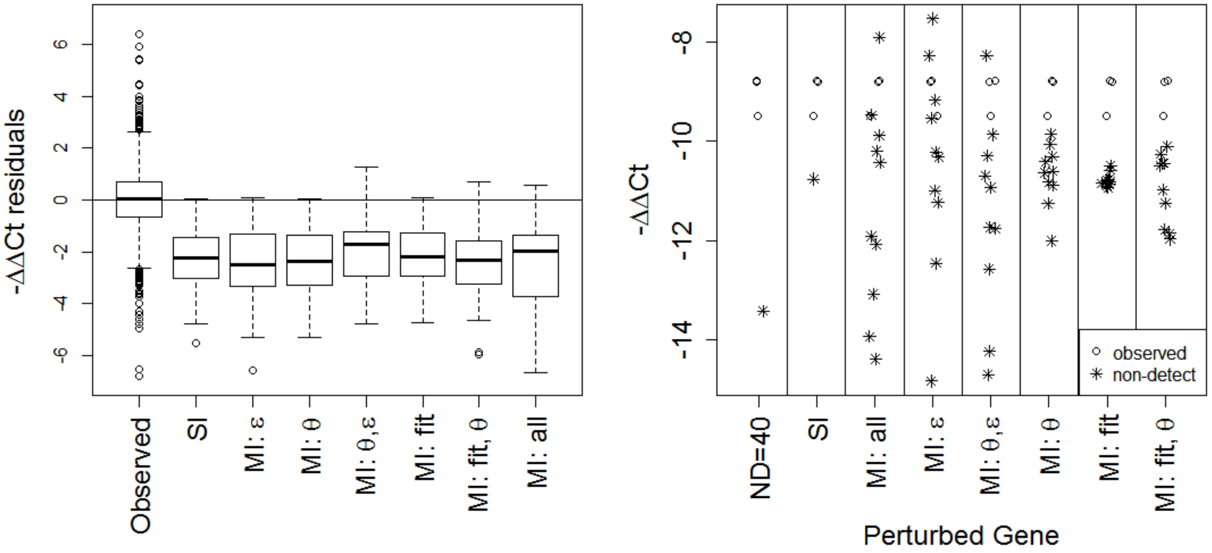

Supplementary Figure 5: Comparison of single and multiple imputation using a real data example. The left panel shows within-replicate residuals stratified by the presence and handling of non-detects. The average ΔΔCt values were calculated over the replicates for gene i and sample j. The residuals for each gene and sample-type were summarized and are plotted here. The box-plots from left to right display the distribution of residuals for: the observed data, missing data after SI, and missing data after MI with different sources of variability. The right panel shows the responses of gene Sema7a to the perturbation of Wnt9a from Dataset 3. Imputed values are denoted with an asterisk. ΔΔCt values produced by replacing a non-detect with a value of 40 are in the leftmost panel, SI estimates are in the next panel, and estimates resulting from applying MI with different combinations of variability sources are shown in the rightmost six panels.

Appendix D - Estimation via Expectation Conditional Maximization (ECM)

Assume Ct values follow a normal distribution, denoted by $y = (x, w)$, where x represents the observed data and w represents the missing data. For a fixed gene $i = 1, ...I$, the gene and sample-type specific mean is denoted by $\theta_{k(j)}$ and σ^2 is a common variance:

$$y \sim N(\theta_{k(j)}, \sigma^2), \text{ and } f(y|\theta_{k(j)}, \sigma^2) = \frac{1}{\sqrt{2\pi\sigma^2}} \exp\Big(-\frac{(y - \theta_{k(j)})^2}{2\sigma^2}\Big).$$

Missing indicator z is a binomial random variable. $z = 1$ if the Ct value is observed and $z = 0$ if the Ct value is missing, so $z_j = 1$ if $y_j = x_j$ observed, and $z_j = 0$ if $y_j = w_j$ is missing.

$$z \sim Bernoulli(p), \text{ and } f(z|p) = p^z(1-p)^{(1-z)}.$$

We model p explicitly as

$$p = \frac{\exp(\beta_0 + \beta_1 y)}{1 + \exp(\beta_0 + \beta_1 y)},$$

where β_0 and β_1 are the coefficients from logistic regression.
The likelihood can be written as:

$$\begin{aligned} L(\theta_{k(j)}, \sigma^2, \beta_0, \beta_1|X, W, Z) &= f(y|\theta_{k(j)}, \sigma^2) f(z|p) \\ &= f(y|\theta_{k(j)}, \sigma^2) f(z|\beta_0, \beta_1, y). \end{aligned}$$

Define $\eta = (\theta_{k(j)}, \sigma^2, \beta_0, \beta_1)$, and

$$\begin{aligned} \log(f(X, W|\eta, Z)) &= \sum_{j=1}^{J} \big[z_j \log(f(x_j|\eta)) + (1 - z_j) \log(f(w_j|\eta))\big], \\ E_W[\log(f(X, W|\eta, Z))] &= \sum_{j=1}^{J} \Big[z_j \log(f(x_j|\eta)) + (1 - z_j) E_W\big[\log(f(w_j|\eta))\big]\Big]. \end{aligned}$$

Furthermore,

$$\begin{aligned} E_W[\log(f(W|\eta, z_j = 0))] &= \int \log(f(w_j|\eta)) f(w_j|\eta, z_j = 0)\, \mathrm{d}w_j \\ &= \int \log(f(w_j|\eta)) \frac{f(w_j, z_j = 0|\eta)}{f(z_j = 0|\eta)}\, \mathrm{d}w_j \\ &= \int \log(f(w_j|\eta)) \frac{f(z_j = 0|w_j, \eta) f(w_j|\eta)}{f(z_j = 0|\eta)}\, \mathrm{d}w_j \\ &= \int \log(f(w_j|\eta)) \frac{f(z_j = 0|w_j, \eta) f(w_j|\eta)}{\int f(z_j = 0|w_j, \eta) f(w_j|\eta)\, \mathrm{d}w_j}\, \mathrm{d}w_j. \end{aligned}$$

The log-likelihood can be written:

$$\begin{aligned} l(\eta|X, W, Z) &= -\frac{J}{2} \log(2\pi) - \frac{J}{2} \log(\sigma^2) \\ &- \frac{1}{2\sigma^2} \Big[\sum_{j=1}^{J} (x_j - \theta_{k(j)})^2 z_j + (w_j - \theta_{k(j)})^2 (1 - z_j)\Big] \\ &+ \sum_{j=1}^{J} \big[z_j \log(f(x_j|\eta)) + (1 - z_j) \log(f(w_j|\eta))\big]. \end{aligned}$$

$$
\begin{aligned}
E_W[l(\eta|\text{rest})] &\propto -\frac{J}{2}\log(\sigma^2) - \frac{1}{2\sigma^2}\Big[\sum_{j=1}^{J}(x_j-\theta_{k(j)})^2 z_j + \sum_{j=1}^{J}E(w_j-\theta_{k(j)})^2(1-z_j)\Big] \\
&+ \sum_{j=1}^{J}\Big[z_j \log\big(\frac{\exp(\beta_0+\beta_1 x_j)}{1+\exp(\beta_0+\beta_1 x_j)}\big) \\
&+ (1-z_j)E_W\big[\log(f(w_j|\eta, z_j=0))\big]\Big] \\
&= -\frac{J}{2}\log(\sigma^2) - \frac{1}{2\sigma^2}\sum_{j=1}^{J}(x_j^2 z_j + E(w_j^2)(1-z_j)) \\
&- \frac{1}{2\sigma^2}\sum_{j=1}^{J}\Big[(-2x_j\theta_{k(j)}+\theta_{k(j)}^2)z_j + (-2E(w_j)\theta_{k(j)}+\theta_{k(j)}^2)(1-z_j)\Big] \\
&+ \sum_{j=1}^{J}\Big[z_j \log\big(\frac{\exp(\beta_0+\beta_1 x_j)}{1+\exp(\beta_0+\beta_1 x_j)}\big) \\
&+ (1-z_j)E_W\big[\log(f(w_j|\eta, z_j=0))\big]\Big].
\end{aligned}
$$

Note $y_j = (x_j, E(w_j))$, and

$$
\text{argmax}_\theta E_W[l(\eta|X,W,Z)] = \text{argmax}_\theta[-\frac{1}{2\sigma^2}\sum_{j=1}^{J}[(-2\theta_{k(j)}y_j + \theta_{k(j)}^2)]].
$$

One can update θ treating $E(w_j)$ as "data", then update σ^2 as:

$$
\frac{1}{J}[\sum_{j=1}^{J}(y_j-\theta_{k(j)})^2 z_j + \sum_{j=1}^{J}(E(w_j^2) - 2\theta_{k(j)}E(w_j) + \theta_{k(j)}^2)(1-z_j)].
$$

Let $\sum_{j=1}^{J}\Big((E(w_j^2) - 2E(w_j)\theta_{k(j)} + \theta_{k(j)}^2)(1-z_j) + (y_j-\theta_{k(j)})z_j\Big) = S$. Then,

$$
\text{argmax}_{\sigma^2} E_W[l(\eta|X,W,Z)] = \text{argmax}_{\sigma^2}\big(-\frac{J}{2}log(\sigma^2) - \frac{1}{2\sigma^2}S\big).
$$

Furthermore,

$$
\begin{aligned}
\text{argmax}_{\underline{\beta}} E_W[l(\eta|X,W,Z)] &= \text{argmax}_{\underline{\beta}}\Big(\sum_{j=1}^{J}\Big[z_j\log\big(\frac{\exp(\beta_0+\beta_1 x_j)}{1+\exp(\beta_0+\beta_1 x_j)}\big) \\
&+ (1-z_j)E_W\big[\log(f(w_j|\eta,z_j=0))\big]\Big]\Big).
\end{aligned}
$$

We perform parameter estimation by using an Expectation Conditional Maximization (ECM) procedure, in this section we outline this process. Let Y be the observed data, X represent the complete data, W denote the non-detects, and Z be a missing data indicator. We model the missing data mechanism as 2 parameter sigmoid function, for example for a logit link:

$$
Pr(Z=1|X) = \frac{\exp(\beta_0+\beta_1\times X)}{1+\exp(\beta_0+\beta_1\times X)}.
$$

Note that the joint $Pr(Z,W) = Pr(W|Z)\times Pr(Z)$, and the marginal probability $Pr(Z) = \int Pr(Z,W)dW$.

The algorithm proceeds as follows:

1. Set initial values of non-detects to a constant, a maximum possible Ct value, say 40. $W = 40$. Obtain the initial estimate of missing data mechanism based on the observed data and initial values of non-detects, $\hat{\beta}_0$ and $\hat{\beta}_1$, using logistic, probit regression, or complementary log-log transformation.

2. Initialize the mean and the variance of the gene expression, $\hat{\theta}$ and $\hat{\sigma}^2$, as sample mean and sample variance

3. E-step 1: Compute $E^{(1)}(W) = \int W \times \dfrac{Pr(Z,W)}{Pr(Z)} dW$.

4. M-step 1: Update $\hat{\theta}^{(1)}$ based on new values of W.

5. E-step 2: Calculate $E^{(1)}(W^2) = \int W^2 \times \dfrac{Pr(Z,W)}{Pr(Z)} dW$.

6. M-step 2: Update $\hat{\sigma}_i^2$, $\hat{\beta}_0$ and $\hat{\beta}_1$. Evaluate the log-likelihood $\log f(\mathbf{Y}|\theta, \sigma, \beta_0, \beta_1)$ based on the new values of W and W^2.

7. Repeat steps 3-6 until the algorithm converges, more specifically until the difference

$$\log f(\mathbf{X}|\theta, \sigma, \beta_0, \beta_1)^{(t+1)} - \log f(\mathbf{X}|\theta, \sigma, \beta_0, \beta_1)^{(t)} < \epsilon,$$

   where ϵ is a tolerance level.
